# A Rare Case of Pyroglutamic Acidosis After Cardiac Surgery

**DOI:** 10.7759/cureus.114932

**Published:** 2026-08-21

**Authors:** Ines Alves, Sara Ranchordas, Marta Marques

**Affiliations:** 1 Cardiac Surgery, Hospital de Santa Cruz, Oeiras, PRT

**Keywords:** 5-oxoproline, cardiac surgery, drug interaction, flucloxacillin, paracetamol, pyroglutamic acidosis

## Abstract

Metabolic acidosis is a common finding following cardiac surgery. Although the differential diagnosis of high-anion-gap metabolic acidosis is well established, less common causes may be overlooked, making the diagnosis challenging.

We report the case of a 59-year-old patient who developed high-anion-gap metabolic acidosis after surgical treatment for mediastinitis, caused by the concomitant use of flucloxacillin and paracetamol. This drug interaction disrupts the γ-glutamyl cycle, leading to the accumulation of 5-oxoproline (pyroglutamic acid). Although rare, this interaction may cause a severe and potentially fatal reaction, particularly in patients with renal failure, sepsis, or malnutrition. As such, the possible diagnosis of pyroglutamic acidosis should be kept in mind in these settings, and the use of these medications should be discontinued immediately.

## Introduction

In the setting of postoperative care after cardiac surgery, metabolic acidosis is a frequent finding and may result from tissue hypoperfusion, cardiogenic shock, renal dysfunction, sepsis, or medication-related adverse effects [1]. It can be influenced by multiple factors, from the patient’s comorbidities to intraoperative and postoperative care.

The differential diagnosis of metabolic acidosis is extensive, and determining whether a normal or elevated anion gap is present helps determine the cause [1]. In cases of an elevated anion gap, frequent causes are hyperglycemia, ketoacidemia, lactic or D-lactic acidosis, intoxication with ethanol, and impaired renal function [1-3]. However, after exclusion of these causes, less frequent etiologies must be considered, and particularly in complex postoperative patients receiving multiple medications, this may present a diagnostic challenge.

Pyroglutamic acidosis is an uncommon but increasingly recognized cause of high-anion-gap metabolic acidosis (HAGMA) [2]. It results from decreased levels of glutathione, leading to a disruption of the γ-glutamyl cycle and the accumulation of 5-oxoproline [4]. One situation that may lead to this is the concomitant and continued use of paracetamol and flucloxacillin, as these medications both trigger the accumulation of 5-oxoproline through different mechanisms [4]. When used together, this combination may cause HAGMA secondary to the accumulation of pyroglutamic acid, particularly in patients with additional risk factors such as sepsis, malnutrition, renal impairment, advanced age, or female sex [2,5,6].

We present the case of a patient who developed HAGMA due to accumulation of pyroglutamic acid in the postoperative period following cardiac surgery, precipitated by the concomitant use of paracetamol and flucloxacillin. Though very rare, this condition is serious and potentially fatal [7].

## Case presentation

A 59-year-old male was admitted to the hospital with a diagnosis of mediastinitis after an uneventful surgical atrial septal defect closure one month prior. Previous relevant history included type 2 diabetes mellitus, hypertension, and atrial fibrillation.

The patient underwent reoperation for surgical debridement of the wound, and closed-suction drains were placed. Wound cultures were positive for methicillin-susceptible Staphylococcus aureus, and treatment was started with flucloxacillin, 2000 mg every four hours. The early postoperative period was uneventful, and the patient was transferred to the ward, where he underwent his course of antibiotics and demonstrated the expected postoperative course and recovery without complications.

On day 37 of hospital admission, the patient presented with disorientation, agitation, dyspnea, and bronchospasm. He was afebrile and tachycardic, with a heart rate of 112 bpm, blood pressure of 92/54 mmHg, respiratory rate of 44 breaths per minute, and oxygen saturation of 99%. Immediate treatment with intravenous corticosteroids, furosemide, and inhaled bronchodilators was administered without improvement. The patient was transferred to an intermediate care unit and started on oxygen at 5 L/min via nasal cannula. Arterial blood gas analysis showed pH 7.13, bicarbonate 5 mEq/L, PCO_2_ 12 mmHg, Na^+^ 140 mEq/L, K^+^ 4.5 mEq/L, Cl^−^ 104 mEq/L, PO_2_ 92 mmHg, lactate 0.9 mmol/L, and glucose 107 mg/dL (Table 1). 

**Table 1:** Initial arterial blood gas analysis PCO₂: partial pressure of carbon dioxide; PO_2_: partial pressure of oxygen; Na^+^: sodium; K^+^: potassium; Cl^-^: chloride.

Parameter	Result	Lab reference value
pH	7.13	7.35-7.45
Bicarbonate	5	21-28 mEq /L
PCO_2_	12	35-45 mmHg
PO_2_	92	75-100 mmHg
Na^+^	140	135-148 mEq/L
K^+^	4.5	3.5–5.0 mEq/L
Cl^-^	104	99-106 mEq/L
Lactate	0.9	0.5-2.0 mmol/L
Glucose	107	70-90 mg/dL

An intravenous sodium bicarbonate infusion of 8.4 g was administered, resulting in a rapid increase in bicarbonate levels and normalization of pH.

Blood analysis showed hemoglobin 7.9 g/dL, leukocytes 14.5 × 10⁹/L, C-reactive protein (CRP) 9.1 mg/dL, urea 36 mg/dL, and creatinine 1.26 mg/dL. The full blood analysis performed is shown in Table 2. Serum ketone levels were within the normal range. Summary transthoracic echocardiography showed no significant changes, excluding pericardial tamponade or cardiac failure. 

**Table 2:** Complete blood analysis

Parameter	Result	Lab reference value
Complete blood count		
Erythrocytes	2.72	4.41-5.90 × 10¹²/L
Hemoglobin	7.9	13.0-17.0 g/dL
Hematocrit	0.239	0.406-0.504 L/L
Mean corpuscular volume	88.1	80.0-96.1 fL
Mean corpuscular hemoglobin	29.3	27.3-33.7 pg
Mean corpuscular hemoglobin concentration	332	328-354 g/L
Red cell distribution width	21.9	11.5-14.5%
White blood cell differential		
Leukocytes	14.5	4.0-10.0 × 10⁹/L
Neutrophils	94.8	40.0-80.0%
Lymphocytes	2.1	20.0-40.0%
Monocytes	2.6	2.0-11.7%
Eosinophils	0.1	1.0-6.0%
Basophils	0.4	0.0-2.0%
Platelets	307	150-400 × 10⁹/L
Clinical chemistry		
Urea	36	13-43 mg/dL
Creatinine	1.26	0.70-1.20 mg/dL
Total bilirubin	0.44	<1.40 mg/dL
Alanine aminotransferase	6	<41 U/L
Alkaline phosphatase	230	40-130 U/L
Gamma-glutamyl transferase	83	10-71 U/L
Sodium	140	136-145mmol/L
Potassium	4.59	3.50-5.10mmol/L
Chloride	103	98-107mmol/L
C-reactive protein	9.1	<0.50mg/dL

The most common causes of high-anion-gap metabolic acidosis (calculated anion gap, 31 mEq/L), such as lactic acidosis, ethanol intoxication, renal failure, and ketoacidosis, were excluded. At this point, the possibility of pyroglutamic acidosis caused by the concomitant prolonged use of flucloxacillin and paracetamol was considered. The patient requested pain control medication frequently, and as a first-line analgesic, paracetamol had been administered routinely during the hospital stay alongside flucloxacillin.

Antibiotic treatment with flucloxacillin had already been stopped the same day, as cultures obtained from the closed-suction drains had shown methicillin-resistant *Staphylococcus epidermidis *and *Citrobacter braakii*, and the antibiotic was escalated to vancomycin and meropenem. Paracetamol was also discontinued and replaced by other analgesics, maintaining effective pain control.

An analysis of serum organic acids was performed, which confirmed the diagnosis of pyroglutamic acidosis, with reported massive levels of pyroglutamic acid of 9960 µmol/mmol creatinine, compatible with pyroglutamic acidosis (Table 3).

**Table 3:** Urine organic acid analysis results

Parameter	Result
Quantitative results	
Pyroglutamic acid (Reference: <63 µmol/mmol)	9960
Qualitative results	
Lactic acid	Moderate quantity
3-Hydroxybutyric acid	Moderate quantity
Acetoacetic acid	Small quantity
2-Hydroxybutyric acid	Small quantity
Adipic acid	Small quantity
4-Hydroxyphenyllactic acid	Small quantity
2-Hydroxyisovaleric acid	Vestigial quantity
Unsaturated adipic acid	Vestigial quantity
2-Octenedioic acid	Vestigial quantity
3-Hydroxydodecanedioic acid	Vestigial quantity
3-Hydroxydodecenedioic acid	Vestigial quantity

After the described changes in treatment, the patient improved, returning to the ward three days later with complete normalization of blood analyses and asymptomatic.

## Discussion

Acquired pyroglutamic acidosis is a rare cause of high-anion-gap metabolic acidosis, with about 100 cases reported since its first description in 1989 [2]. Although infrequent, the diagnosis should be suspected and treatment initiated, as it is potentially fatal [2,6]. It results from the accumulation of pyroglutamic acid and is frequently linked to the use of paracetamol and, in several cases, the concomitant administration of flucloxacillin [2,7].

Flucloxacillin is a beta-lactam antibiotic, a semisynthetic isoxazolyl penicillin, used to treat Gram-positive bacterial infections, mainly *Streptococcus* and *Staphylococcus *species. It is commonly used for the treatment of susceptible skin and soft tissue infections [6]. Therefore, flucloxacillin is frequently used in infected surgical wounds.

Effective analgesia is one of the key factors that strongly influences functional recovery in the postoperative period. Paracetamol is frequently a first-line analgesic after surgical procedures, including cardiac surgery. Consequently, it is not uncommon for both medications to be used at the same time in the setting of post-cardiac surgery.

**Figure 1:**
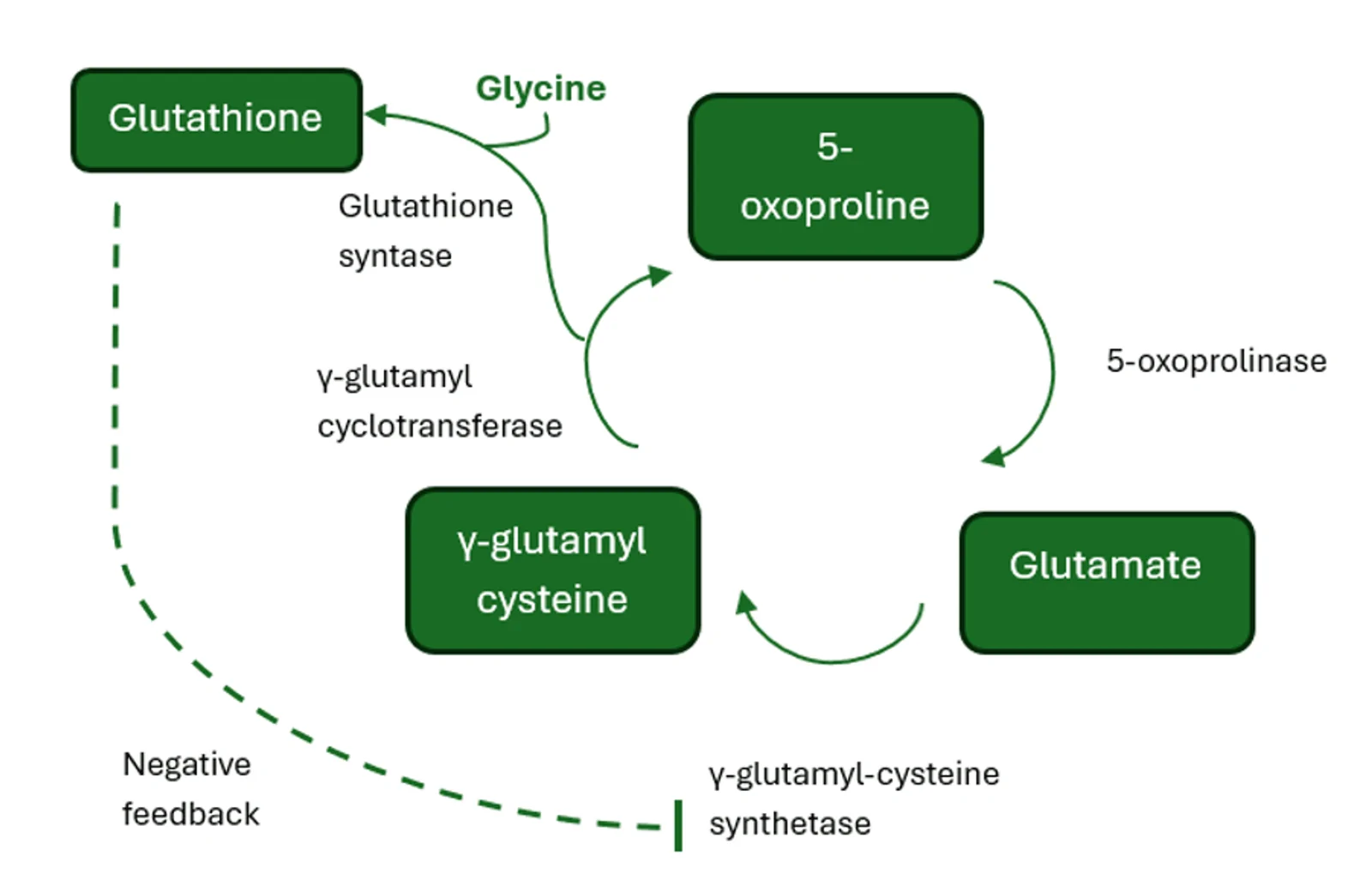
γ-glutamyl cycle This figure was created using Microsoft Word (Microsoft Corporation, Redmond, Washington) [4].

When used together, these medications can cause a rare interaction in which acidemia results from the accumulation of pyroglutamic acid, also known as 5-oxoproline, which is a natural amino acid derivative that acts as an intermediate of the γ-glutamyl cycle (Figure 1) [4]. Pyroglutamic acid dissociates into H^+^ and pyroglutamate. It is an anion at physiological pH and, therefore, an increase in this unmeasured anion leads to high-anion-gap metabolic acidosis [4]. 

**Figure 2:**
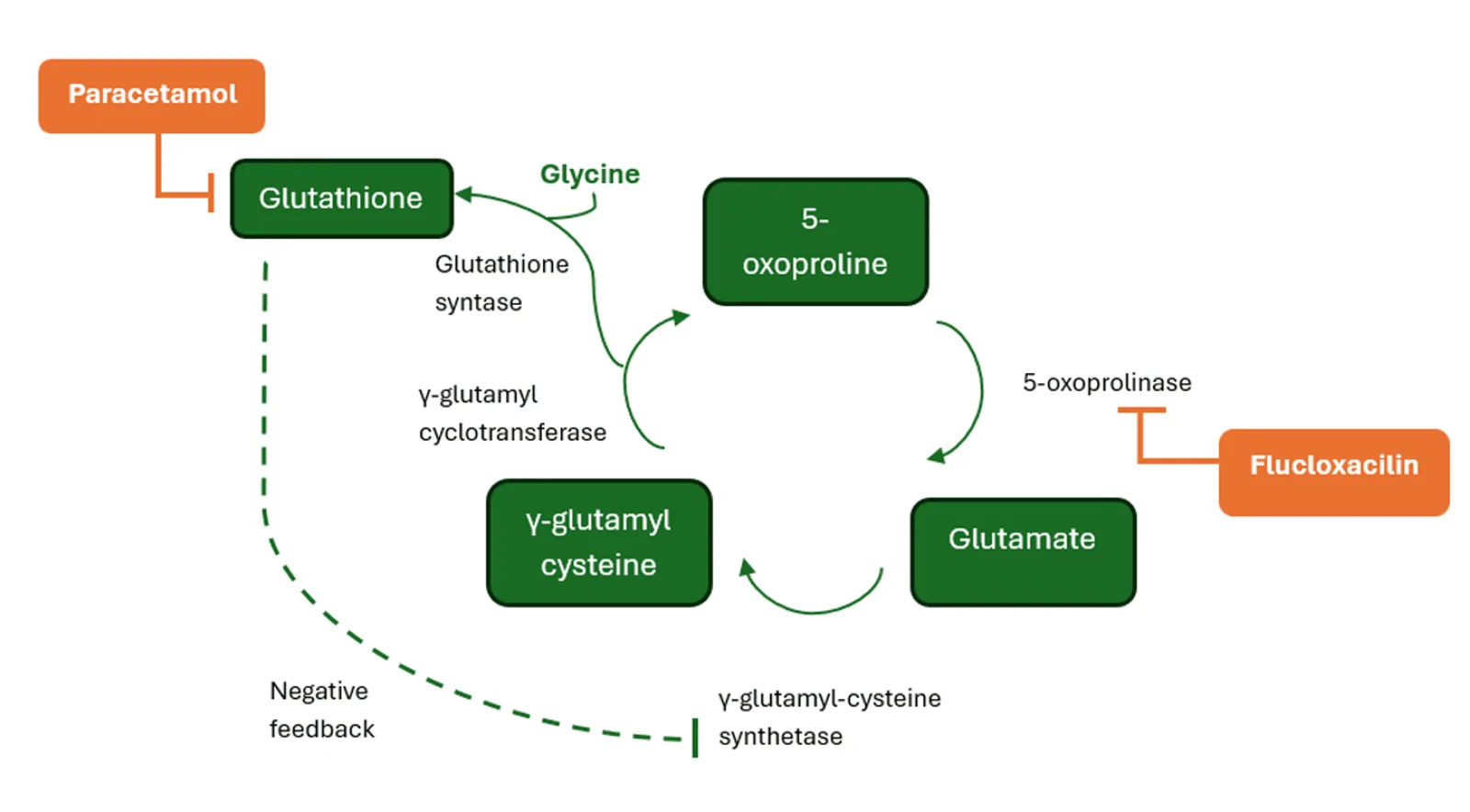
Effects of paracetamol and flucloxacillin in the γ-glutamyl cycle This figure was created using Microsoft Word (Microsoft Corporation, Redmond, Washington) [4].

Flucloxacillin and paracetamol favor the increase in 5-oxoproline through different mechanisms (Figure 2) [4]. Paracetamol is metabolized into N-acetyl benzoquinoneimine, which forms an irreversible bond with glutathione, decreasing its levels, especially with high doses or long-term use [4]. The decreased levels of glutathione lead to a lack of negative feedback, resulting in increased activity of the enzyme γ-glutamyl-cysteine synthetase and, therefore, increased production of γ-glutamyl-cysteine, which is converted into 5-oxoproline [4]. On the other hand, flucloxacillin inhibits 5-oxoprolinase, the enzyme responsible for metabolizing 5-oxoproline into glutamate, leading to the accumulation of 5-oxoproline (Figure 2) [4]. 

Therefore, concomitant use of paracetamol and flucloxacillin results in the accumulation of 5-oxoprolinase through increased production due to the depletion of glutathione and decreased elimination by inhibition of its conversion to glutamate [4].

Concomitant risk factors are renal insufficiency (since clearance of 5-oxoproline occurs partially via renal excretion), sepsis, and malnutrition [6]. By contributing to glutathione deficiency, advanced age, female sex, chronic liver failure, and alcohol abuse may also increase the risk of pyroglutamic acidosis [2,5].

Diagnosis is often made based on drug history, risk factors, and arterial acid-base analysis [7]. The anion gap is crucial, and diagnosis is made by exclusion of more common causes of high-anion-gap acidosis [2,7]. For definitive diagnosis, urine or blood can be sent for pyroglutamate measurement [2,8]. The results may not be available for several days, so treatment should be initiated in the meantime [2].

Treatment involves supportive care, with fluids and bicarbonate if appropriate, stopping paracetamol, and changing flucloxacillin to another antibiotic [2,8]. In severe cases, dialysis or hemofiltration should be considered [2]. N-acetylcysteine (NAC) has been used successfully in some cases and has a good safety profile [2,9].

Our patient was treated with flucloxacillin and paracetamol for a long period of time, resulting in the accumulation of pyroglutamic acid. Pyroglutamic acidosis is usually a diagnosis of exclusion [3]. Given the presence of high-anion-gap metabolic acidosis and the absence of other identifiable causes, the possibility was considered. Treatment consisted of discontinuation of the drugs that caused the metabolic acidosis, with resolution of blood analysis alterations and symptoms. Later, the diagnosis was confirmed by the presence of an increased serum concentration of pyroglutamate.

## Conclusions

Although rare, the concomitant administration of paracetamol and flucloxacillin may cause a severe and potentially fatal reaction, particularly in patients with renal failure, sepsis, or malnutrition. Pyroglutamic acidosis is a probably underdiagnosed cause of metabolic acidosis with increased anion gap, and it is important to keep this possibility in mind. In these patients, acid-base balance should be monitored, and paracetamol should be discontinued immediately.
